# The Prussian Blue Reaction of the Liver in Pernicious Anæmia

**Published:** 1907-11-02

**Authors:** 


### THE PRUSSIAN BLUE REACTION OF THE LIVER IN PERNICIOUS ANÆMIA.

It is sometimes very difficult to be sure that a patient has been suffering from pernicious anæmia, notwithstanding examinations during life. The pathognomonic point about the blood is the high colour index in the presence of marked anæmia, a point we have already discussed; but owing to personal and instrumental errors of technique it not infrequently happens that there is some uncertainty as to what the patient's blood-colour index really is; moreover, it occasionally happens that an inquest has to be held in a case where no blood examination has been made during life. It is then important to know how to recognise pernicious anæmia after death.

It may be urged, of course, that there are many different types of severe and apparently causeless anæmia, each of which merits the term "pernicious"; but there is no doubt that, even at the expense of leaving some such cases nameless at present, there is one particular type which is clinically so different to the others that it merits a name to itself, and it is to this type that the term "pernicious anæmia" is generally restricted. It is not our purpose to enlarge upon the clinical symptoms of this disease, for they are well known—the primrose-yellow skin, the marked inability for exertion, the absence of considerable loss of subcutaneous fat, the slight pyrexia, the hæmic bruits, the palpable liver, the absence of marked enlargement of spleen or lymphatic glands, the tendency to hæmorrhages (purpuric, retinal, nasal, etc.), the tendency to severe gastro-intestinal symptoms particularly diarrhœa, the likelihood of one or more remissions under arsenical treatment, and the certainty of ultimate complete asthenia, and death. Our point is that, notwithstanding all the above, it is often uncertain even at the autopsy whether pernicious anæmia proper was the real diagnosis.

Of all the post-mortem findings, that which is most pathognomonic is the Prussian blue reaction of the liver. The method of performing the test is as follows:—Two solutions are prepared. The first is a 5 per cent. solution of potassium ferrocyanide in water; the other a 1 per cent. solution of hydrochloric acid. A thin slice of the liver is cut, and immersed in the potassium ferrocyanide for five minutes; it is then lifted out and transferred directly into the weak hydrochloric acid. In a case of pernicious anæmia the surface of the slice of liver begins to go bright blue in two minutes, and the blue goes on increasing for twenty minutes or half an hour. The reaction is, of course, conversion of the iron in the liver into ferric-chloride by the hydrochloric acid, and the conversion of the ferric-chloride into Prussian blue by the potassium ferrocyanide. The liver may only go a sort of pale green; that is not a positive reaction, and the condition is not one of pernicious anæmia. The blue must be a real blue, and a fairly deep one; on looking carefully at the stained liver, it will be seen that the blue is deepest around the periphery of each lobule, so that a fine tracery or network of blue is apparent on close inspection. In the case of the spleen the blue reaction is not so constant, though when it does occur it may be universal: in the case of the kidney it is particularly the glomeruli that react, giving the appearance of fine dots of deep blue all through the cortex. It is a very pretty reaction, pathognomonic, and very easy to obtain if ordinary care is observed.

